# The Splice of Life: Does RNA Processing Have a Role in HIV-1 Persistence?

**DOI:** 10.3390/v13091751

**Published:** 2021-09-02

**Authors:** Alexander O. Pasternak, Ben Berkhout

**Affiliations:** Laboratory of Experimental Virology, Department of Medical Microbiology, Amsterdam UMC, University of Amsterdam, 1105 AZ Amsterdam, The Netherlands; b.berkhout@amsterdamumc.nl

**Keywords:** RNA processing, splicing, HIV-1 persistence, HIV-1 reservoir, HIV-1 latency

## Abstract

Antiretroviral therapy (ART) suppresses HIV-1 replication but does not eradicate the virus. Persistence of HIV-1 latent reservoirs in ART-treated individuals is considered the main obstacle to achieving an HIV-1 cure. However, these HIV-1 reservoirs are not transcriptionally silent, and viral transcripts can be detected in most ART-treated individuals. HIV-1 latency is regulated at the transcriptional and at multiple post-transcriptional levels. Here, we review recent insights into the possible contribution of viral RNA processing to the persistence of HIV-1 reservoirs, and discuss the clinical implications of persistence of viral RNA species in ART-treated individuals.

## 1. Introduction

In infected individuals who adhere to combination antiretroviral therapy (ART), HIV-1 replication is potently and durably suppressed, which restores the immune function and prevents the development of AIDS [1]. However, ART is not curative and has to be sustained lifelong. HIV-1 forms long-lived reservoirs in infected individuals, which persist despite decades of suppressive ART and fuel viral rebound if therapy is interrupted. The persistence of viral reservoirs is the main obstacle to achieving an HIV-1 cure [2,3,4,5]. Latent infection of resting CD4+ T cells and possibly some other cell types, such as macrophages, is thought to be the main mechanism of HIV-1 persistence in peripheral blood and lymphatic tissues [6,7]. Traditionally, HIV-1 latency has been viewed primarily as transcriptional latency (replication-competent transcriptionally silent proviruses that can be reactivated to transcribe viral RNA and produce infectious virus). However, virus latency does not require a complete shutdown of viral gene expression, only a lack of infectious progeny production, and latency can be regulated not only at the transcriptional but also at multiple post-transcriptional levels (e.g., splicing and nuclear export of viral RNA, translation into viral proteins, virus particle assembly and maturation) [7,8]. Indeed, cell-associated (CA) HIV-1 RNA can be detected in the majority of peripheral blood samples from HIV-infected individuals on prolonged ART in the absence of ex vivo stimulation [9,10,11,12,13]. Furthermore, experimental administration of latency reversal agents (LRAs) to infected individuals on ART, designed to “shock-and-kill” the virus, has frequently resulted in an increase in CA HIV-1 RNA transcripts and sometimes plasma viremia, but this has rarely translated into a reduction in the viral reservoir [14,15,16,17]. Although the interpretation of the results of such trials is complicated by the large excess of genetically defective over replication-competent proviruses in ART-treated individuals [18,19,20], it is plausible that current LRA drugs are unable to completely reverse HIV-1 latency. Therefore, for the design of more effective therapeutic interventions, it is important to better understand the mechanisms that control HIV-1 persistence. Here, we discuss some recent insights into the possible role of viral RNA processing, particularly splicing, in the persistence of HIV-1 reservoirs.

## 2. A Brief Overview of HIV-1 Splicing

**Figure 1:**
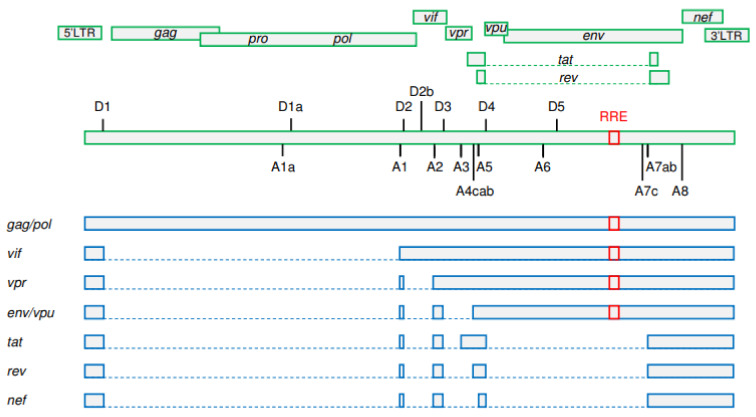
A simplified schematic representation of HIV-1 splicing. Upper panels: HIV-1 open reading frames. Positions of donor (D1–D5) and acceptor (A1–A8) splice sites and of the Rev response element (RRE) are shown. Lower panels: the main representatives of unspliced, incompletely spliced, and multiply spliced HIV-1 RNA classes. Gag and Pol proteins are expressed from the 9 kb unspliced RNA. The other HIV-1 proteins are expressed from either incompletely (Vif, Vpr, Env, and Vpu) or multiply (Tat, Rev, and Nef) spliced RNAs. RRE is present in unspliced and incompletely spliced RNAs but not in multiply spliced RNAs. Exons are shown by bars and introns by dashed lines. (Figure adapted from [24]; reproduced, with permission, from Elsevier).

More than 100 different viral transcripts can be detected in HIV-infected cells, all derived by alternative splicing from full-length unspliced (US) primary HIV-1 RNA transcripts that are transcribed from the integrated provirus (Figure 1) [21,22]. As HIV-1 splicing has been covered in depth by several recent reviews [23,24,25], we will only provide a very brief overview here. Upon proviral integration, only short (2 kb) completely spliced, also called multiply spliced (MS), transcripts are initially produced that encode the regulatory proteins Tat, Rev, and Nef. In general, cells do not tolerate nuclear export of intron-containing RNA molecules, which are either completely spliced and exported or degraded in the nucleus. However, HIV-1 has evolved a mechanism that allows efficient nuclear export of US and incompletely spliced viral RNA transcripts. As the infection progresses, a shift can be observed towards the production of 9 kb US and 4 kb incompletely spliced transcripts that encode the structural and accessory proteins Gag, Pol, Env, Vif, Vpr, and Vpu [26,27]. This shift is dependent on a threshold level of the Rev protein, which facilitates the export of the US and incompletely spliced RNA from the nucleus by binding to the Rev response element (RRE), a stem-loop structure located in the *env* open reading frame of these RNAs [25,28,29]. Importantly, this temporal shift from the production of MS to the production of US RNA was observed not only during in vitro HIV-1 infection but also after stimulation of latently infected cell lines [30,31,32].

## 3. US/MS RNA Ratio as a Clinical Biomarker

In the pre-ART era, the relative dynamics of US and MS RNA in untreated HIV-infected individuals was studied by a number of laboratories. Several groups reported an association between the US/MS RNA ratio and disease progression: higher US/MS RNA ratios were measured in typical/rapid progressors, while slow progressors and long-term nonprogressors were characterized by low ratios [33,34,35,36,37]. In view of the temporal shift from MS to US RNA production discussed above, a higher US/MS RNA ratio in an infected individual might reflect a higher frequency of HIV-infected cells in the later stages of the viral replication cycle, which is characterized by elevated expression of viral proteins and production of virus particles. Such cells could exert pressure on the host immune system, causing persistent immune activation and thus contributing to rapid disease progression. Interestingly, it was recently shown that viral protein expression is not even necessary for US RNA to cause immune activation because intron-containing HIV-1 RNA can induce innate immune signaling in diverse cell types, such as dendritic cells, macrophages, and CD4+ T cells [38,39,40].

Remarkably, we recently demonstrated that the same US/MS RNA ratio, measured at 12 weeks of ART, was negatively predictive of the immunological response to ART (absolute and relative CD4+ T-cell count) at 48 and 96 weeks of therapy, outperforming a number of immunological biomarkers of T-cell activation, exhaustion, and apoptosis [41]. Moreover, the US/MS RNA ratio was positively associated with markers of CD4+ T-cell activation and apoptosis at 12 weeks of ART [41]. At first glance, the fact that the same biomarker is associated with similar clinical endpoints in untreated and treated infection seems puzzling because HIV-1 biology, as well as the state of host immunity, is very different in an untreated versus a treated infection. By stopping virus replication, ART strongly selects against productively infected cells, and in a latently infected cell population, the US/MS RNA ratio is not supposed to have the same meaning as in an untreated infection. However, the decay of HIV-infected cells after ART initiation is multiphasic [42,43], with short-lived cells dominating the total infected cell pool during the first months of ART. A study estimated that at 12 weeks of ART 90% of infected cells are labile, which masks the persistent HIV-1 reservoir [44]. These cells are likely different from the long-lived reservoir cells that support latent infection. In particular, the activation level of these cells may be higher, and therefore they can impose a less severe block to productive infection than the long-lived reservoir cells. The US/MS RNA ratio in such cells can reflect the relative number of productively infected cells that either directly influence the subsequent immune reconstitution on ART or reflect the impaired state of host immunity that contributes to the immunological failure. Also, as discussed above, US RNA can induce immune activation on its own, without the need for protein expression. This implies that even some defective proviruses can have this activity if the Rev–RRE axis remains intact [38], providing another possible mechanism for residual pathogenesis of defective proviruses [45,46]. Importantly, the fact that a virological biomarker performed better than any immunological biomarker in predicting an immunological outcome highlights the importance of considering the residual HIV-1 activity on ART as a possible cause of the residual immune dysfunction that frequently occurs despite virologically suppressive ART.

## 4. US and MS RNA in the HIV-1 Reservoir

HIV-1 splicing is relatively inefficient, even in a productive infection, due to the relative weakness of the viral splice sites, except D1 and D4 [24,47]. These suboptimal signals are thought to be necessary to ensure that splicing can be regulated, and that the HIV-1 RNA is not overspliced, such that proper amounts of US and incompletely spliced RNAs are synthesized to drive a productive infection cycle. Due to this, and to the mechanism of nuclear export of US RNA, US RNA significantly outnumbers incompletely spliced and MS RNA in both untreated and treated infection [8,10,11,41,48,49,50]. Moreover, MS RNA decays much faster and to a larger extent than US RNA upon ART initiation [41,51,52,53]. As a result, the US/MS RNA ratios and the US RNA+/MS RNA+ cell ratios are much higher in treated than in untreated individuals ([41], our unpublished observations). Although US RNA in ART-treated individuals is readily detectable [8,54], it is challenging to detect MS RNA in individuals on long-term ART without ex vivo cellular stimulation [54,55,56]. One reason behind this disproportion can be a previously underappreciated latency block to splicing, recently proposed by the Yukl group [8]. Their work has challenged the dogma that HIV-1 latency is mainly regulated at the level of transcriptional initiation, as it revealed several additional reversible blocks to transcriptional elongation, polyadenylation, and splicing [8,57]. Interestingly, ex vivo TCR stimulation results in a much more prominent increase in MS RNA compared to US RNA, both in ART-treated individuals and in primary cell models [8,58]. Here it must be noted that several commonly used LRAs, despite stimulating US RNA transcription, are unable to induce MS RNA expression [8,55,59], confirming the existence of a single or multiple additional blocks to splicing. Some members of the minor spliceosome pathway were differentially expressed between unstimulated and activated cells from primary cell models and ART-treated individuals [58], suggesting that these genes may play a role in the splicing block.

However, even upon full stimulation, MS RNA levels remain much lower than US RNA levels, and MS RNA+ cells remain much rarer than US RNA+ cells [8,60]. In part, this might be due to the timing of ex vivo stimulation experiments, as usually only one post-stimulation time point is assessed. As discussed above, early studies repeatedly observed a temporal shift from the production of MS to US RNA after stimulation of latently infected cell lines [30,31,32]. While these early studies used quantitative assays that were clearly inferior to the current techniques, for a full understanding of HIV-1 latency and reactivation it might be informative to assess US and MS RNA at several time points post-stimulation. Another potential reason behind the large difference between the US and MS RNA levels even after latency reversal, mostly relevant to infected cells from ART-treated individuals, is that these cells frequently harbor proviruses with defects in the HIV-1 regulatory protein Tat [61,62]. While Tat is required for high-level viral transcription, studies have demonstrated that it also enhances HIV-1 splicing [63,64,65]. Whereas some US RNA can still be produced by Tat-independent transcription from defective proviruses harboring deletions or other defects in the *tat*/*rev* region, the absence of Tat can impose an additional block to the production of spliced HIV-1 RNA species. In addition, splicing requires the presence of several intact genomic regions, such as splice sites of exonic splicing enhancers. Therefore, US RNA+ cells probably harbor defective proviruses more frequently than MS RNA+ cells, and the MS RNA transcription competence may be a more proximal surrogate of proviral replication competence compared to the US RNA transcription competence [55,56,66]. This is also supported by the recent observation that upon ex vivo LRA treatment, MS RNA but not US RNA correlated with HIV-1 RNA in the culture supernatant (the latter assay measures virus release upon latency reversal) [55]. Nevertheless, several groups reported that US RNA, measured at ART interruption, predicts the time to viral rebound [54,67,68,69], and we recently demonstrated that the pre-treatment interruption level of US RNA was independently predictive not only of the time to viral rebound, but also of the magnitude of the viral rebound [54]. This suggests that despite being partly composed of defective proviruses, the US RNA ‘transcription-competent reservoir’ does reflect the replication-competent reservoir [4,70,71].

In addition to the genuine lower copy numbers of MS RNA as compared to US RNA, MS RNA is also more challenging to detect in infected individuals by qPCR-based or digital PCR-based approaches due to primer/probe-template mismatches that occur more frequently with MS RNA assays. The reason for this difference is that any MS RNA-specific amplicon (ideally, the probe) should span the D4-A7 exon-exon junction, which strongly constrains the primer and probe design to two very short regions just 5′ of D4 and 3′ of A7. These regions are quite heterogeneous in sequence, even within one HIV-1 subtype, thus resulting in primer/probe-template mismatches. In contrast, the US RNA-specific assay design is much less constrained in terms of the genomic region targeted, allowing one to select highly conserved regions with much lower sequence heterogeneity. This means that the chance of under-quantitation (or even the generation of a false negative result) due to primer/probe-template mismatches is higher for MS than for US RNA. It is important to realize that not all primer/probe-template mismatches, even if a small deletion at the target site is present, are due to defective proviruses and in many cases the provirus will still be intact despite harboring a sequence variation at the primer/probe binding site. However, the differences between US and MS RNA copy numbers have been measured even when patient-matched primers and probes were used, arguing that detection issues only partially contribute to the observed differences in abundance between the US and MS HIV-1 RNA species [10,72].

## 5. Splicing between HIV-1 and Host Genes: A Win-Win Situation?

HIV-1 latent reservoirs are believed to persist primarily by cell longevity and proliferation [73]. Studies reported a significant enrichment of proviral integrations in genes that are involved in cellular proliferation and survival, such as *BACH2*, *MKL2*, *STAT5B*, and others, in cells from ART-treated individuals [74,75,76]. In theory, such integrations can lead to the clonal expansion of the infected cell, which would favor viral persistence. One mechanism by which a provirus integrated in the same orientation as the local host transcription unit may modulate host gene expression is aberrant splicing that produces virus-host chimeric mRNAs. Such chimeras have the potential to alter cellular gene expression and could even lead to proliferation of the host cell [77,78]. Indeed, such chimeric transcripts, mostly resulting from splicing between the HIV-1 D4 splice donor site and a human splice acceptor site, have been detected by several groups, either by RNA sequencing approaches [20,79,80] or by targeted RT-PCR to amplify specific virus-host transcripts [81]. These chimeric mRNAs were found both during in vitro HIV-1 infection of primary human T cells [20,79] and in CD4+ T cells from ART-treated individuals, with or without ex vivo stimulation [80,81]. By CRISPR-mediated approaches, Liu et al. demonstrated that this aberrant host gene expression can be driven by the HIV-1 LTR promoter [80]. Thus, HIV-1 integration in specific, proliferation-related, loci may create a win-win situation for the virus and the host cell, in which the virus provides a strong promoter that, by means of aberrant splicing, alters host gene expression in a way that ensures both cellular proliferation and viral persistence.

If such aberrant splicing patterns are indeed beneficial to the persistence of both the provirus and the infected host cell, then one would expect them to be selected with time on ART, especially in expanded cell clones. Such in vivo selection has yet to be demonstrated, but Pinzone et al. observed that, with time, ART-treated individuals select for defective proviruses that retain the D4 splice donor site [20]. The same study, by RNA sequencing of in vitro infected CD4+ cells, reported that aberrant splicing occurs 20-fold more frequently between D4 and downstream host exons in comparison to the D1 major splice donor site [20]. A similar bias towards the use of D4 in the virus–host chimeric spliced transcripts was observed by Sherrill-Mix et al. [79]. One explanation of this preferential use of D4 is that A7, the viral splice acceptor site downstream of D4, is relatively weak [24,47], such that it can easily be outcompeted by stronger splice acceptor sites in the flanking cellular RNA sequences. Because splicing between D4 and A7 is a necessary step for MS RNA production, this implies that aberrant virus–host splicing events may also cause a decrease in HIV-1 MS RNA levels. In theory, this may be one of the reasons behind low MS RNA copy numbers in ART-treated individuals and primary cell models, even after ex vivo stimulation [58]. Further studies should access the role of this aberrant splicing in HIV-1 persistence, but if it interferes with genuine HIV-1 splicing, in particular with MS RNA production, in ART-treated individuals, then it can be one of the factors that contribute to the state of “deep latency”, the situation where a genetically intact provirus cannot be reactivated to induce a productive infection [82,83]. To answer this question, technically demanding single-cell assays will be necessary.

## 6. Other Steps in HIV-1 RNA Processing

Not much is currently known about a possible role of other HIV-1 RNA processing steps such as capping, polyadenylation, and nuclear export in viral persistence. Yukl et al. [8] observed lower copy numbers of polyadenylated HIV-1 RNA compared to elongated transcripts (long LTR, *pol*, *nef*), but it is unclear whether this reflects a specific latency block to polyadenylation or simply a gradual decrease in the transcript copy number with increasing transcript size, due to premature termination of transcription during elongation [84,85]. MS RNA has been shown to be retained in the nucleus both in resting CD4+ T cells from individuals on ART and in a chemokine-induced model of HIV-1 latency in primary resting CD4+ T cells [86,87]. This nuclear localization of MS RNA precluded high-level transcription and nuclear export of other CA HIV-1 RNA species and protein translation and possibly contributed to the viral latency. This block to nuclear export of MS RNA could be overcome by overexpression of HIV-1 Tat or the polypyrimidine tract binding protein, which triggered virus release [86]. In addition, differential expression of Rev cofactors, such as MATR3 or PSF, can contribute to the block in nuclear export of HIV-1 RNAs [88,89,90]. In J-Lat cells, LRA-induced HIV-1 p24 protein expression was abolished by a shRNA-mediated MATR3 knockdown and could be rescued by ectopic expression of MATR3 [91]. Effects of different LRAs on the expression of MATR3 in ex vivo stimulated peripheral blood cells from ART-treated individuals corresponded to their effects on reactivation of HIV-1 US RNA and cell-free viral RNA expression [91]. Other host factors that are differentially expressed in different cell types and individuals can, in theory, lead to variations in the HIV-1 splicing pattern, as well as in other steps of viral RNA processing, between cell types and/or infected individuals. However, experimental evidence for this is still lacking [23].

## 7. Conclusions

Although HIV-1 latency is clearly regulated at both transcriptional and post-transcriptional steps of viral gene expression, it is still unclear whether RNA processing has a critical role in the persistence of the viral reservoirs. HIV-1 has evolved weak splice sites to allow export of US and incompletely spliced RNA species, but this may also lead to aberrant virus–host splicing events. If such aberrant splicing leads to changes in the expression of cellular genes involved in cellular proliferation, this can enhance HIV-1 persistence in expanded cellular clones. At the same time, these virus–host splicing events can inhibit the use of A7 splice acceptor site, and therefore suppress MS RNA production, potentially inducing a state of “deep latency”. In light of the recent efforts to develop new therapeutic interventions that could induce a “blocked and locked” state of HIV-1 proviruses [92,93], HIV-1 splicing and RNA processing in general could be a potential therapeutic target. Yeh et al. recently reported that filgotinib, an FDA-approved JAK inhibitor, specifically suppressed HIV-1 splicing, as well as aberrant HIV-driven host gene expression [94]. This inhibitor and other recently identified drugs that target viral transcription or RNA processing [95,96,97,98] have the potential to suppress virus-induced immune activation and dysfunction, facilitate immune reconstitution on ART, and possibly contribute to the “functional cure” of HIV-1.
